# Transanal total mesorectal excision of giant villous tumor of the lower rectum with McKittrick–Wheelock syndrome: a case report of a novel surgical approach

**DOI:** 10.1186/s40792-019-0728-0

**Published:** 2019-11-06

**Authors:** Masahiko Fukase, Hiroshi Oshio, Sho Murai, Tomomi Kawana, Yusuke Saito, Emiko Kono, Yukiko Oshima, Gen Yunome, Shin Teshima, Masaaki Ito

**Affiliations:** 1 Department of Surgery, Sendai Medical Center, 2-11-12 Miyagino, Miyagino-ku, Sendai, Miyagi-ken 983-8520 Japan; 2 Department of Colorectal Surgery, National Cancer Center Hospital East, 6-5-1 Kashiwanoha, Kashiwa-shi, Chiba-ken 277-8577 Japan

**Keywords:** Transanal total mesorectal excision, Villous tumor, McKittrick–Wheelock syndrome, Lower rectal cancer, Total mesorectal incision

## Abstract

**Background:**

McKittrick–Wheelock syndrome (MKWS) is caused by a villous tumor of the rectosigmoid colon with hypersecretion of mucus containing electrolytes. Complete resection of the tumor is needed to cure this disease. Transanal total mesorectal excision (TaTME) is currently a promising treatment for lower rectal tumor because of the reliability of its resection margin especially in bulky tumor. We present this first case report of a TaTME for MKWS with a lower rectal tumor.

**Case presentation:**

An 81-year-old woman was admitted to our hospital with diarrhea and acute renal failure. Computed tomography and magnetic resonance imaging examinations revealed an 80-mm-sized enhanced tumor located in her lower rectum without lymph node swelling and distant metastasis. A giant villous tumor secreting mucus was seen in the lower rectum to the anal canal during colonoscopy. The result of tumor biopsy was adenocarcinoma. To preserve the anal function and ensure distal margin, we chose TaTME for curative resection. After improving the electrolyte imbalance, TaTME was performed successfully and R0 resection was achieved. There was no sign of recurrence or electrolyte depletion for 1 year after the surgery.

**Conclusion:**

TaTME could be a promising surgical approach for giant villous tumor with MKWS in the lower rectum.

## Introduction

McKittrick–Wheelock syndrome (MKWS) was first reported as a rare syndrome characterized by dehydration, electrolyte depletion, and renal failure due to secretory diarrhea from villous tumor of the rectosigmoid colon [1]. This tumor not only causes systemic disease, but also has a risk of malignancy due to its size. The definitive treatment is partial colectomy followed by fluid and electrolyte replacement. An endoscopic approach is a limited option because of its circumference, malignancy, and location [2–4]. Especially in the case of tumors located in the lower rectum, we cannot avoid choosing abdominal–peritoneal resection, which reduces the patients’ quality of life drastically. The technique is sometimes too invasive compared with the risk of malignancy.

Transanal total mesorectal excision (TaTME) is a newly prevalent surgical technique for middle and lower rectal cancer. TaTME has the advantage of obtaining TME and quality of life through visualization of the distal part of the surgical plane especially in case of bulky tumor [5, 6]. Here, we present a first case report of a female patient with MKWS successfully treated by TaTME.

## Case presentation

**Fig. 1:**
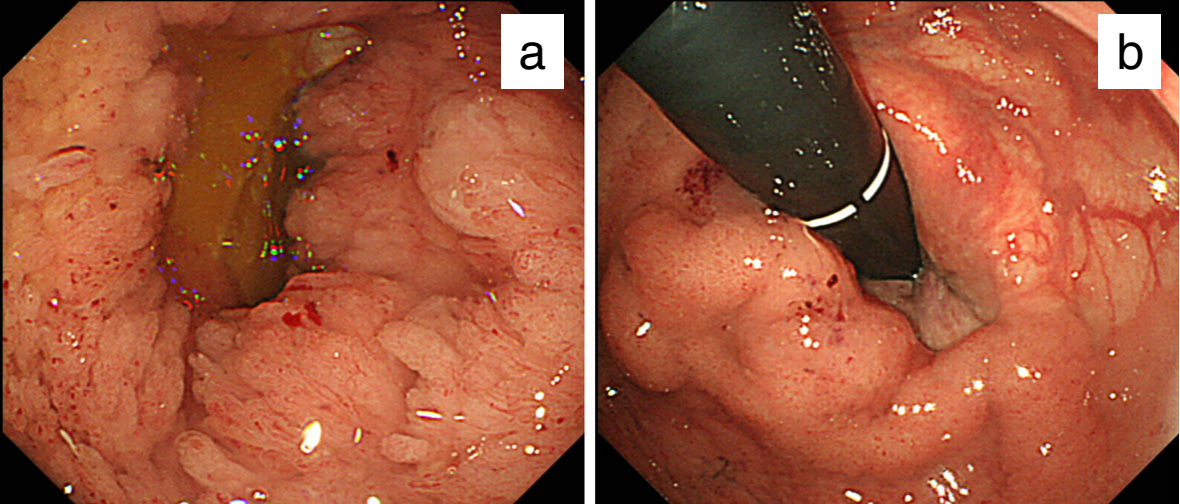
Colon fiber findings. **a** A giant villous tumor with rich secretion of mucus was found in the lower rectum. **b** The tumor located from the anal canal to 10 cm on the oral side

**Fig. 2:**
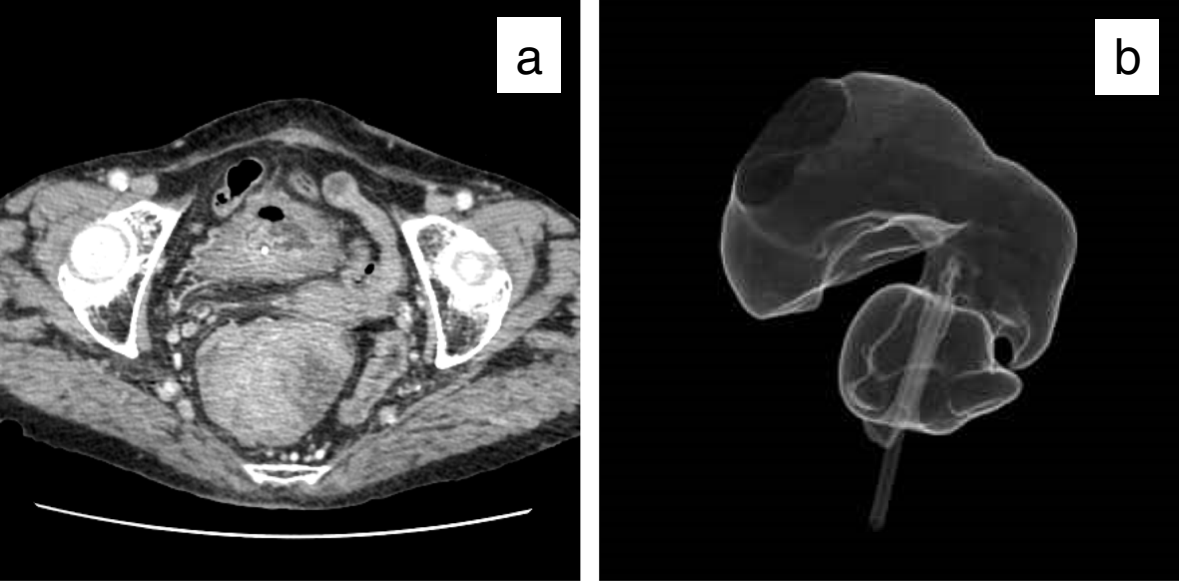
Computed tomography findings. **a** Enhanced tumor was found in the lower rectum without lymph node metastasis. **b** The computed tomography colonography showed that the tumor extended into anal canal

**Fig. 3:**
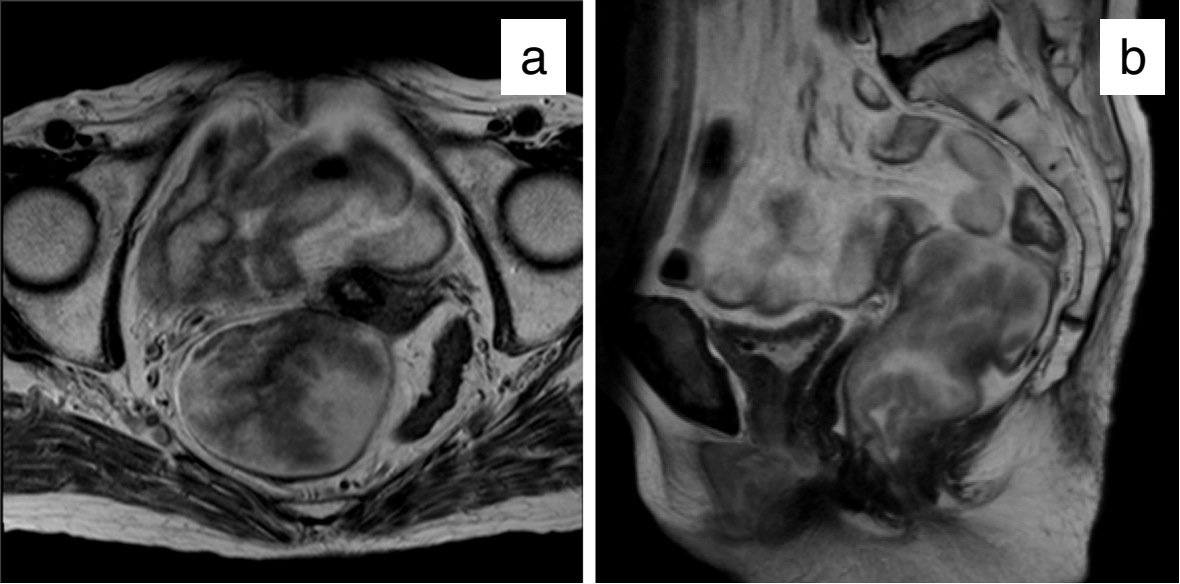
Magnetic resonance imaging findings. **a** Axial. **b** Sagittal. There was no sign of invasion up to the vagina and anal sphincter

An 81-year-old woman was admitted to our hospital suffering from general fatigue and severe diarrhea, which contained a lot of mucus. On physical examination, she did not have abdominal pain with normal vital sign. Laboratory data showed acute renal failure (creatinine, 3.07 mg/dL; normal range, 0.47 to 0.79 mg/dL) with hyponatremia (sodium, 109 mEq/L; normal range, 139 to 146 mEq/L), hypokalemia (potassium, 3.6 mEq/L; normal range, 3.7 to 4.8 mEq/L), and hypochloremia (chloride, 66 mEq/L; normal range, 101 to 109 mEq/L). However, the collected mucus contained a high concentration of electrolytes (sodium, 159 mEq/L; potassium, 14.1 mEq/L; chloride, 140 mEq/L). Elevation of the tumor markers carcinoembryonic antigen and carbohydrate antigen 19-9 was not detected. Digital rectal examination revealed huge circumferential mass of the rectum from 4 cm from anal verge. So we performed a colonoscopy because electrolyte depletion from mucus was considered as a cause of renal dehydration. The lower rectum was occupied by a giant villous tumor located from Hermann’s line to 10 cm on the oral side. The tumor with rich secretion of mucus encompassed the full circumference of the rectum (Fig. 1a, b). Multiple tumor biopsy showed well-differentiated adenocarcinoma cells in some lesion of the tumor. An enhanced mass, 8 cm in diameter located in the lower rectum without lymph node and distant metastases, was found in a computed tomography (CT) scan (Fig. 2a). The CT colonography showed that the tumor was located in the lower rectum with extending into the anal canal (Fig. 2b). Magnetic resonance imaging showed no sign of infiltration of the vagina and anal sphincter (Fig. 3a, b).

The patient was diagnosed with a villous adenocarcinoma with MKWS because of the typical findings for the villous tumor, such as electrolyte and body fluid depletion with secretory diarrhea. After intensive fluid therapy, tumor resection was required. Transanal endoscopic microsurgery and endoscopic mucosal dissection were excluded because of its circumference and extension. Considering the patient’s quality of life and tumor location up to Hermann’s line, we chose TaTME as a radical treatment.

**Fig. 4:**
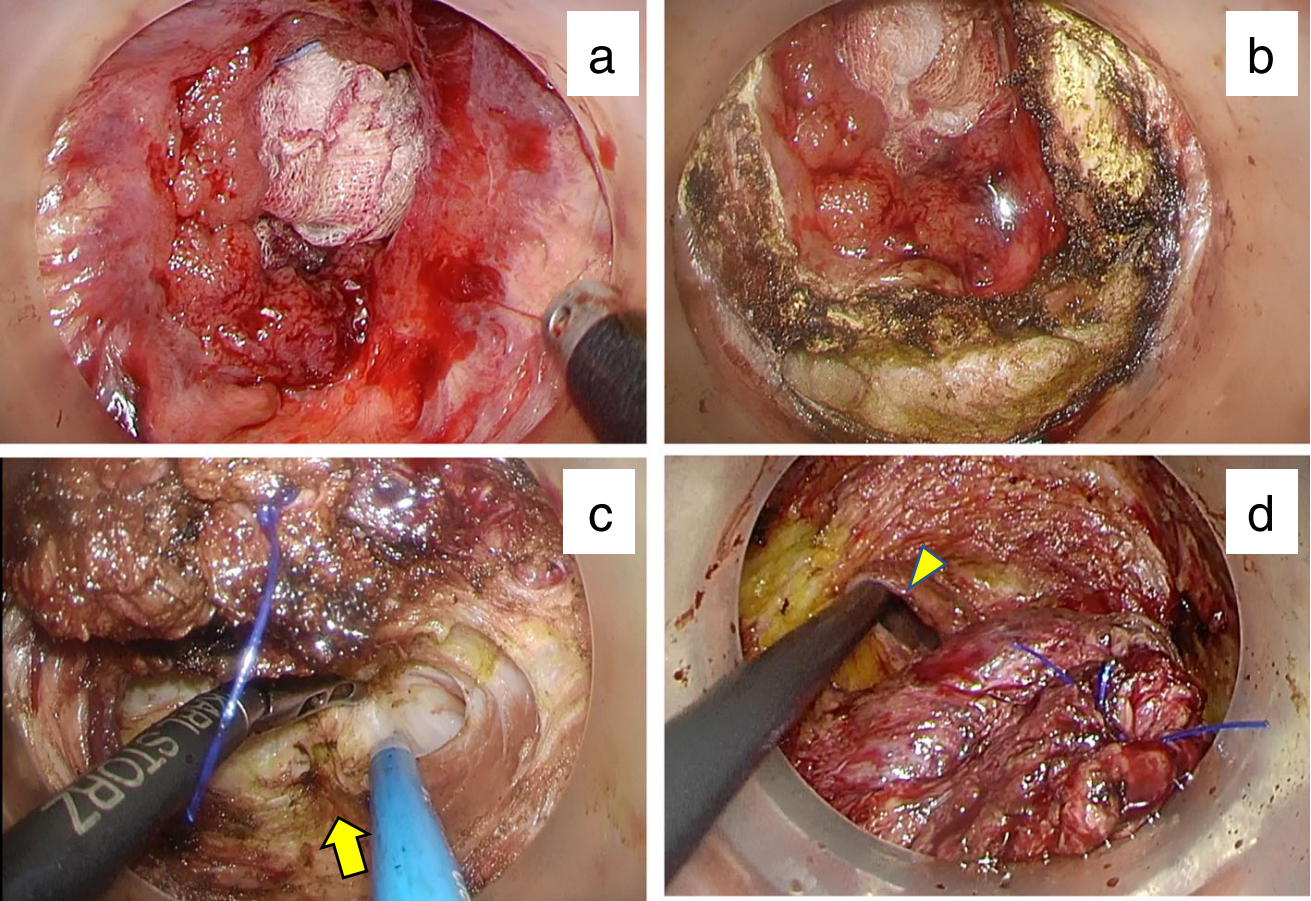
Intraoperative views of transanal approach. **a** Distal edge of the tumor. **b** Circumferential mucosectomy. **c** Hiatal ligament (arrow). **d** Abdominal cavity from transanal approach (arrowhead)

We performed TaTME with one team and anal approach first as described below. First, a Lone Star Retractor (Lone Star Medical Products, Houston, TX, USA) was inserted under general anesthesia in the lithotomy position. We used the GelPOINT Path Transanal Access Platform (Applied Medical, Rancho Santa Margarita, CA, USA) and AirSeal (ConMed, Utica, NY, USA) as an insufflation system to obtain a stable pneumorectum with smoke evacuation. We pushed the tumor away with gauze because the collapsed giant tumor and a huge amount of mucus interrupted the laparoscopic view. The tumor was located on a hemorrhoid 4 cm distant from the anal verge (Fig. 4a). At first, circumferential mucosectomy was performed with a 5-mm distal margin from the tumor because its malignancy potential was not as high (Fig. 4b). Then, the lumen of the rectum was closed with a purse-string suture to prevent cancer cell dissemination and mucus leakage. Endopelvic fascia was identified after intersphincteric resection of the muscle. After dissection of the hiatal ligament, the TME plane was revealed in transanal approach (Fig. 4c). The abdominal cavity was opened at the level of the peritoneal reflection (Fig. 4d). Thus, we inserted five ports and transferred to a traditional laparoscopic TME technique. The inferior mesenteric artery and vein were highly ligated after full mobilization of the left and sigmoid colon with connection to TME plane. A small laparotomy at the umbilical port site was required for extraction of the specimen because the tumor was too bulky. We pulled the stump of the oral colon out anally and performed hand-sewn coloanal anastomosis. Additionally, we constructed a diverting loop ileostomy on the right lower port site. The operative time was 281 min, and the blood loss was 50 mL.

**Fig. 5:**
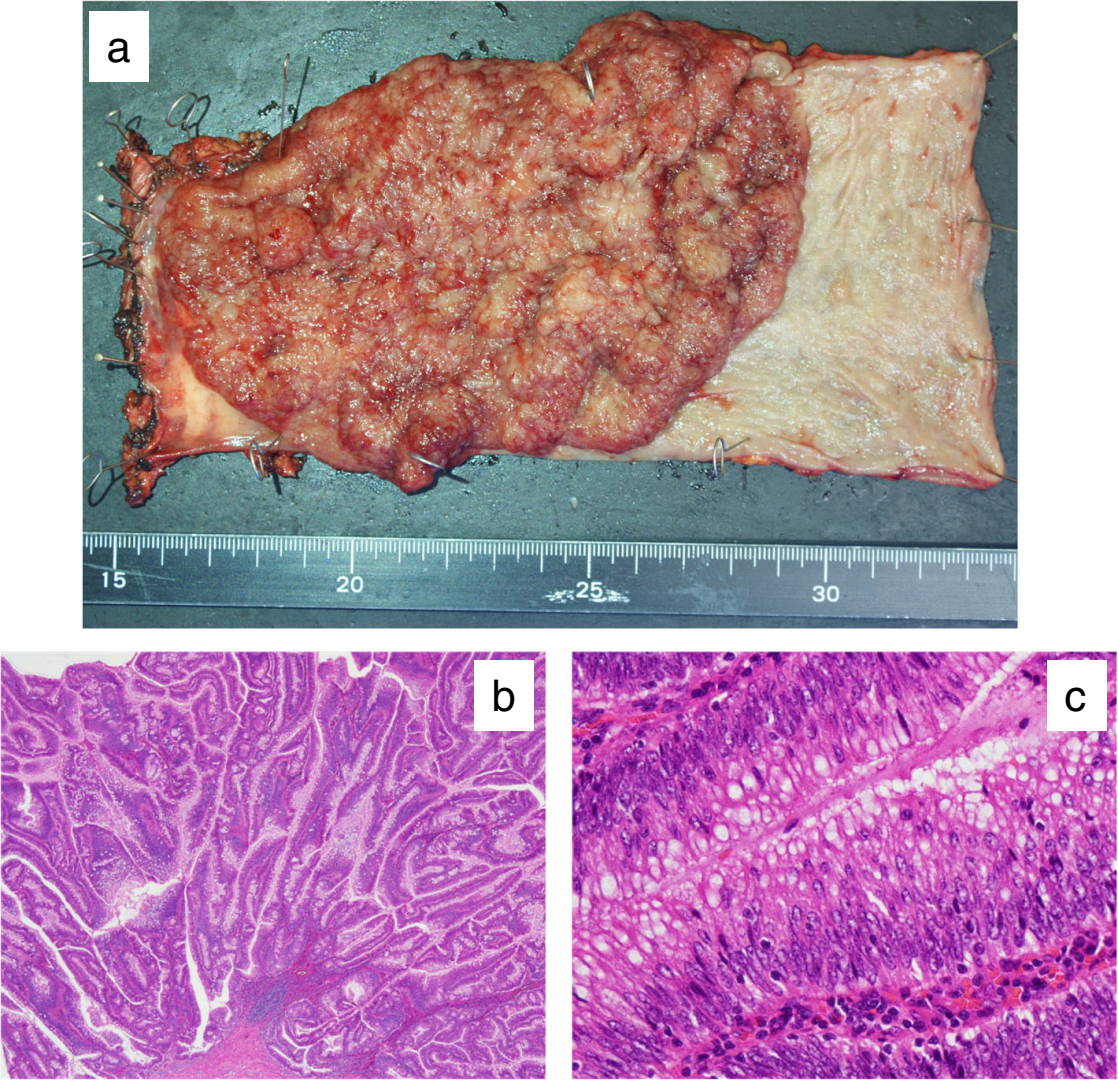
Surgical specimen and microscopic findings. **a** A villous tumor at gross examination. **b** Hematoxylin–eosin stain (× 20) revealed papillary tumor. **c** Differentiated adenocarcinoma in tubulovillous adenoma was detected in some lesion (× 200)

On gross examination, the tumor was a 13.5-cm papillary tumor. The distal margin was more than 5 mm away from the tumor (Fig. 5a). The histological findings showed an intramucosal adenocarcinoma in the tubulovillous adenoma located in the lower rectum without regional lymph node metastasis (Fig. 5b, c). The cancer classification was Tis, N0, M0, stage 0 in Union for International Cancer Control 8th edition. The patient was discharged 14 days after surgery in good general conditions with no postoperative complications, including anastomotic trouble. There was no evidence of recurrence, and we closed the covering ileostomy 5 months after surgery. She had good defecation function and remained well at 12 months after the first surgery. Adjuvant chemotherapy and radiotherapy were not performed, and the patient recovered full activities of daily living.

## Discussion

The MKWS shows hyponatremia, hypokalemia, and hypochloremia because of the loss of a high amount of mucus in the patient’s diarrhea. Its triad was chronic mucus diarrhea, renal function impairment with hydroelectrolyte imbalance, and giant rectosigmoid tumor. The distal location of the tumor prevents reabsorption of abnormally secreted electrolyte in normal colonic mucosa. This is a rare disease, which occurs only in 0.27–2.4% of villous adenomas representing 3–6% of colonic tumors [2, 7–9]. The size of tumors with MKWS is usually over 3–4 cm in diameter. According to a review of 64 reported cases of MKWS, the maximum diameter was 4–26 cm (mean, 13.9 cm). Giant tumors often contain high-grade dysplasia, and 72.4% of tumors with MKWS were diagnosed as carcinoma [2, 10].

The primary treatment is fluid therapy for renal failure before curative therapy. Some conservative therapies were reported in addition to invasive therapies including surgery. Nonsteroidal anti-inflammatory agents, such as cyclooxygenase-2 inhibitor, contribute to improve severe dehydration and renal failure through reduction of the stool output [11, 12]. Endocavity irradiation was tried in 19 patients with villous adenoma, which resulted in 32% recurrence [13]. However, mortality from untreated syndrome was reported in 100% [14]. Therefore, removal of the tumor is the only radical treatment for this syndrome.

Among the less-invasive treatments, transanal minimally invasive surgery and transanal endoscopic microsurgery were performed. These were challenging and difficult procedures in giant tumor even in experienced hand. Rectal stricture treated with multiple times dilation was reported as postoperative complication in case of 16-cm villous tumor with MKWS [15, 16]. As endoscopic approach, endoscopic submucosal dissection was reported, even in tumors over 20 cm in size. However, it always resulted in piecemeal resection because the tumor often covered 100% of the circumference [17, 18]. Most importantly, these less invasive therapies without TME are not radical therapy for the cases of T1 adenocarcinomas.

According to previous reports, laparoscopic lower anterior resection and proctosigmoidectomy are the most frequently applied transabdominal surgical procedures for MKWS [8, 15, 19–21]. The summary of these previous reports is shown in Table 1. The procedures could lead to severe complication such as anastomotic leakage, urinary dysfunction, and surgical site infection [15, 19–24]. Especially in the case of tumors located in the lower rectum, we cannot avoid choosing abdominal–peritoneal resection, which reduces the patient’s quality of life dramatically [10, 25]. The technique is sometimes too invasive; thus, we focused on TaTME as a less-invasive anal-preserving surgery.

**Table 1:** Patients with McKittrick-Wheelock syndrome treated with laparoscopic low anterior resection and proctosigmoidectomy

Author	Year	Age, sex	Tumor size (cm)	Circumference (%)	Distance from anal verge	Procedure	Pathology
Dagan and Reissman [8]	2010	52, F	31	100	On the dentate line	LPS+TM+DLI	TVA, HGD
Podesta et al. [19]	2014	72, F	15	100	On the dentate line	LAR	VA, HGD
Choi et al. [20]	2012	59, M	25	100	7 cm	LAR	TVA
Targarona et al. [21]	2008	63, F	18	ND	< 1 cm	LAR	VA, HGD
		69, F	16		1 cm	IPS	VA
		69, F	7		10 cm	LAR	VA, HGD

*DLI* diverting loop ileostomy, *HGD* high-grade dysplasia, *IPS* intersphincteric proctosigmoidectomy, *LAR* laparoscopic low anterior resection, *ND* not described, *TM* transanal mucosectomy, *TVA* tubulovillous adenoma, *VA* villous adenoma

TaTME is a relatively new surgical approach for rectal tumors, which was introduced by Lacy and Adelsdorfer [26]. TaTME can make up for the shortcomings of laparoscopic TME using a transanal laparoscopic platform based on the concept of bottom to top approach. The clinical benefits of TaTME have been reported, such as longer resection margin, low circumferential margin positive rates, less morbidity, and more sphincter-saving rectal resections. Fernández-Hevia et al. reported TaTME had shorter operative time, less readmission, and 1.1 cm longer distal margin compared to laparoscopic TME [27–29]. The technique has advantages in the cases of narrow pelvis, bulky tumors, and fatty mesorectum. There were some reports of large rectal tumor such as leiomyosarcoma and gastrointestinal stromal tumors occupying the pelvis removed successfully with this approach [6, 30, 31].

In our case, we discuss the surgical approach to this tumor from the perspectives of curability and quality of life. It is very difficult to accomplish lower anterior resection of the rectum because the huge tumor spread to the anal canal and disturb the laparoscopic view. Abdominal perineal resection would be too invasive for this elderly patient because of her Tis or T1 tumor, although the surgical margin would certainly be obtained. Thus, we chose TaTME as a radical anal-preserving surgery. Purse-string suture could completely prevent flow of plentiful mucus into laparoscopic view. As planned, negative surgical margin was obtained without any complication. This is a first report of a patient of MKWS successfully treated with TaTME.

## Conclusion

Complete resection in this case of giant villous tumor with MKWS was successfully achieved by TaTME without loss of anal function. TaTME could be a promising surgical approach for a villous tumor with MKWS in the lower rectum.

## Data Availability

The dataset supporting the conclusions of this article is included in the article.

## Abbreviations

CT: Computed tomography
MKWS: McKittrick–Wheelock syndrome
TaTME: Transanal total mesorectal excision
